# Prevalence and Severity of Cognitive Impairment Among Older Adults Using Benzodiazepines in Primary Care: Association with Duration of Use and Prescribing Pattern in Mexico

**DOI:** 10.3390/healthcare14131916

**Published:** 2026-07-01

**Authors:** Roberto Mariano-Ramírez, Osmar Antonio Jaramillo-Morales, María Teresa de la Garza-Carranza, Josué Vidal Espinosa-Juárez, Nereida Violeta Vega-Cabrera, Jaime Isael Flores-Rosas, Tomás Valdivieso-Nieves, Juan Ramón Ruíz-Carlo

**Affiliations:** 1 Departamento de Medicina y Nutrición, División de Ciencias de la Salud, Campus León, Universidad de Guanajuato, León 37320, Guanajuato, Mexico; lirlok90@hotmail.com; 2 Coordinación Clínica de Educación e Investigación en Salud, HGZMF 2, Instituto Mexicano del Seguro Social, Irapuato 36650, Guanajuato, Mexico; 3 Departamento de Enfermería y Obstetricia, División de Ciencias de la Vida, Campus Irapuato-Salamanca, Universidad de Guanajuato, Irapuato 36500, Guanajuato, Mexico; nv.vega@ugto.mx; 4 Departamento de Ciencias Económico Administrativas, Tecnológico Nacional de México en Celaya, Antonio García Cubas Pte. 1200 Esq. Ignacio Borunda, Celaya 38010, Guanajuato, Mexico; teresa.garza@itcelaya.edu.mx; 5 Escuela de Ciencias Químicas, Universidad Autónoma de Chiapas, Ocozocoautla de Espinosa 29140, Chiapas, Mexico; josue.espinosa@unach.mx; 6 Coordinación Clínica de Cirugía, HGZMF 2, Instituto Mexicano del Seguro Social, Irapuato 36650, Guanajuato, Mexico; zrewolf@gmail.com; 7 Coordinación Clínica de Educación e Investigación en Salud, HGZ 4, Instituto Mexicano del Seguro Social, Celaya 38060, Guanajuato, Mexico; tomas.valdivieso@imss.gob.mx

**Keywords:** benzodiazepines, cognitive impairment, aged, primary health care, mini-mental state examination

## Abstract

**Highlights:**

**What are the main findings?**

- Longer duration of benzodiazepine use in older adults was associated with higher prevalence of cognitive impairment in a primary care population.
- Greater cognitive impairment was observed with increasing duration of benzodiazepine use in crude analyses.

**What are the implications of the main findings?**

- Findings should be interpreted as exploratory and descriptive, given the observational and unadjusted nature of the analyses.
- Careful prescribing, regular medication review, and consideration of deprescribing strategies remain important in older adults.

**Abstract:**

**Background:** Benzodiazepines are widely prescribed to older adults in primary care despite concerns regarding their potential cognitive adverse outcomes. Evidence on the association between benzodiazepine use and cognitive impairment remains inconsistent and is limited in middle-income countries, particularly in routine primary care settings. **Objectives:** To evaluate the association between benzodiazepine use and cognitive impairment among older adults in a primary care hospital in Mexico and to explore clinical factors associated with benzodiazepine prescribing patterns. **Methods:** A cross-sectional analytical study was conducted using non-probabilistic convenience consecutive sampling in a sample of 228 in adults aged ≥60 years attending a primary care hospital in Irapuato, Mexico, during 2025. Cognitive status was assessed using the Mini-Mental State Examination (MMSE) and classified as normal cognition, mild/moderate cognitive impairment, or major cognitive impairment. Benzodiazepine exposure was identified through pharmacy records and categorized by duration of exposure (<3 years, 3–6 years, and >6 years). Sociodemographic variables were collected. Associations were evaluated using chi-square tests, and crude logistic regression models were used to estimate odds ratios (ORs) with 95% confidence intervals (CIs). An additional exploratory analysis assessed clinical factors associated with benzodiazepine type using a dichotomized outcome to ensure model stability. **Results:** A total of 228 older adults with documented benzodiazepine use were included. The sample represents the complete population of eligible benzodiazepine users during the study period. Cognitive impairment was identified in 61.8% of participants. Longer duration of benzodiazepine use was significantly associated with worse cognitive status (χ^2^ = 25.81; *p* < 0.001), with the highest proportion of major impairment observed in individuals with more than six years of use. In exploratory analyses, benzodiazepine prescribing patterns were significantly associated with sleep disorders (χ^2^ = 11.56; *p* = 0.009), polypharmacy (χ^2^ = 10.28; *p* = 0.016), and MMSE category (χ^2^ = 13.87; *p* = 0.031). In adjusted models, polypharmacy was associated with increased odds of receiving the most frequently prescribed benzodiazepine (OR 2.37; 95% CI 1.27–4.41; *p* = 0.007), while sleep disorders (OR 2.10; 95% CI 1.10–3.90; *p* = 0.009) and cognitive status (OR 1.85; 95% CI 1.05–3.20; *p* = 0.031) also showed significant associations. No comparisons with non-users were performed due to lack of comparable cognitive assessment; however, this comparison should be interpreted cautiously due to differences in assessment methods between groups. **Conclusions:** Among benzodiazepine users in primary care, longer duration of exposure was associated with worse cognitive status. Additionally, prescribing patterns were influenced by clinical factors such as polypharmacy, sleep disorders, and cognitive status, suggesting a predominantly clinically driven approach. These findings highlight benzodiazepine use as an important marker of cognitive vulnerability and underscore the need for careful prescribing, regular medication review, and deprescribing strategies in older adults.

## 1. Introduction

Population aging has led to a substantial increase in the prevalence of cognitive impairment worldwide, making it a major public health concern. Cognitive impairment in older adults ranges from mild cognitive impairment to major neurocognitive disorders and is associated with functional decline, reduced quality of life, increased healthcare utilization, and mortality [1,2]. In primary care settings, early identification of cognitive impairment is particularly relevant, as these services often represent the first point of contact for older adults experiencing cognitive changes. In this context, brief cognitive screening instruments, such as the Mini-Mental State Examination (MMSE), remain widely used in primary care due to their feasibility and long-standing clinical acceptance; however, their diagnostic performance and interpretation depend strongly on clinical context and population characteristics [3,4].

Pharmacological exposure represents one of the most important and potentially modifiable factors associated with cognitive impairment in later life. Among commonly prescribed medications, benzodiazepines warrant particular attention. These agents are frequently used in older adults for the management of anxiety, insomnia, and other neuropsychiatric conditions, especially in primary care. Due to age-related pharmacokinetic and pharmacodynamic changes, older individuals are more vulnerable to adverse outcomes associated with benzodiazepine use, including sedation, psychomotor impairment, delirium, falls, and cognitive dysfunction [5,6]. Consequently, benzodiazepines are classified as potentially inappropriate medications for older adults in the American Geriatrics Society Beers Criteria^®^, which recommend avoiding their routine use when possible [7].

Despite these recommendations, benzodiazepine prescribing remains common in routine clinical practice. Numerous observational studies have examined the association between benzodiazepine use and cognitive impairment or dementia, with heterogeneous results. Several systematic reviews and meta-analyses have reported significant associations between benzodiazepine exposure and impaired cognitive performance or increased risk of dementia [8,9,10], whereas others have suggested weaker associations after accounting for confounding factors such as anxiety, sleep disorders, or comorbidities [9,10]. Importantly, evidence increasingly indicates that the duration of exposure and cumulative use play a central role, with long-term benzodiazepine use being more consistently associated with adverse cognitive outcomes than short-term exposure [9,11].

Recent longitudinal and neuroimaging studies have provided additional insight into this association. Population-based cohorts have reported associations between benzodiazepine use and structural brain changes, including reduced hippocampal and thalamic volumes and accelerated brain atrophy over time [12]. Although these findings do not establish causality, they support the hypothesis that benzodiazepine use may be associated with subclinical or early cognitive vulnerability in aging populations.

Most of the available evidence on benzodiazepine use and cognitive impairment originates from high-income countries and specialized clinical settings [8,9,10]. Differences in prescribing practices, healthcare access, educational attainment, and social and cultural contexts limit the generalizability of these findings to middle-income countries [5,7]. In Mexico, benzodiazepines remain widely prescribed in primary care [13]; however, population-based evidence examining their association with cognitive impairment in older adults within routine clinical practice is scarce.

Given this context, the present study aimed to evaluate the association between benzodiazepine use, including duration of exposure, and cognitive impairment among adults aged 60 years and older attending a primary care hospital in Mexico. By providing real-world data from a routine primary care setting, this study seeks to contribute to a better understanding of cognitive vulnerability associated with benzodiazepine use and to inform safer prescribing and medication review practices in older adults. This study is particularly relevant given the lack of real-world evidence in middle-income countries and the need to better understand cognitive vulnerability among benzodiazepine users in primary care. Rather than evaluating causal relationships, this study focuses on describing patterns of cognitive impairment within this population.

## 2. Materials and Methods

### 2.1. Study Design and Setting

This was a cross-sectional analytical study conducted during 2025 in a primary care unit, Hospital General de Zona con Medicina Familiar (HGZ MF No. 2) of the Mexican Institute of Social Security (IMSS), located in Irapuato, Guanajuato, Mexico. This institution provides first-level healthcare services to an urban and semi-urban population and serves as a referral center for preventive, chronic, and geriatric care.

### 2.2. Study Population

The study population consisted of adults aged 60 years or older who were registered beneficiaries of the hospital. Eligible participants were those with documented use of at least one benzodiazepine medication during routine medical care and who agreed to participate in the study. Benzodiazepine use was verified through pharmacy dispensing records from the hospital’s pharmacy department.

#### 2.2.1. Inclusion Criteria

- Age ≥ 60 years.
- Registered beneficiary of the HGZ MF No. 2.
- Current or previous use of benzodiazepines documented in pharmacy records.
- Ability to undergo cognitive evaluation.
- Provision of written informed consent.

#### 2.2.2. Exclusion Criteria

- Older adults without a history of benzodiazepine use.
- Individuals with acute delirium, severe sensory deficits (visual or auditory), or medical conditions that could interfere with reliable cognitive assessment at the time of evaluation.
- Incomplete clinical or cognitive assessment data.

### 2.3. Sample Size and Sampling

All identified older adults meeting the inclusion criteria during the study period were invited to participate. 228 participants were included using consecutive sampling. All eligible benzodiazepine users during the study period (2025) were included (census approach. Participants were recruited consecutively during routine outpatient visits, reflecting real-world clinical practice and reducing selection bias. A formal sample size calculation was not performed because all eligible patients during the study period were included (census approach).

### 2.4. Variables and Data Collection

Data were collected through structured interviews and review of clinical and pharmacy records during outpatient visits in family medicine and geriatrics clinics.

#### 2.4.1. Sociodemographic Variables

The following characteristics were recorded:

- Age (categorized as 60–69, 70–79, 80–89, and ≥90 years).
- Sex.
- Educational level (illiterate, primary school, secondary school, high school, or university degree).

#### 2.4.2. Benzodiazepine Exposure

Benzodiazepine use was characterized according to:

- Type of benzodiazepine prescribed.
- Duration of use, categorized as:
  <3 years
  3–6 years
  >6 years
- Pharmacokinetic profile of the benzodiazepine (short-, intermediate-, or long-acting), based on standard classifications.

Benzodiazepine exposure was defined based on pharmacy dispensing records and may include both current and previous use at the time of cognitive assessment. This approach reflects real-world prescribing patterns but does not allow distinction between active and past use.

#### 2.4.3. Assessment of Cognitive Status

Cognitive function was assessed using the Mini-Mental State Examination (MMSE) [3], a widely used and validated instrument for screening cognitive impairment. The MMSE evaluates multiple cognitive domains, including orientation, registration, attention and calculation, recall, language, and visuospatial abilities.

The instrument consists of 30 items, with a total possible score ranging from 0 to 30 points, where higher scores indicate better cognitive function. Cognitive status was classified according to established cut-off points, considering normal cognition (24–30 points), mild/moderate cognitive impairment (≤23 points), and severe cognitive impairment (≤17 points), adjusted for educational level when applicable.

The MMSE was administered by trained personnel under standardized conditions to ensure consistency and reliability in data collection. The MMSE has demonstrated good validity and reliability in older adult populations [14,15].

MMSE scores were interpreted considering participants’ educational level. However, no formal statistical adjustment or score transformation was applied. In participants with low educational attainment or illiteracy, MMSE results were interpreted cautiously due to known limitations of the instrument in these populations.

Information on selected clinical variables, including polypharmacy, sleep disorders, and alcohol use, was available from clinical records and incorporated into exploratory analyses. However, these variables were not originally collected using standardized instruments, and therefore were considered secondary in the analytical framework. These variables were analyzed to better characterize prescribing patterns in real-world clinical practice.

### 2.5. Statistical Analysis

Data were entered into an anonymized database and analyzed using IBM SPSS Statistics version 25 (IBM Corp., Armonk, NY, USA). Descriptive statistics were used to summarize sociodemographic characteristics, benzodiazepine exposure, and cognitive status.

Categorical variables were expressed as frequencies and percentages. Associations between benzodiazepine use duration and cognitive impairment were evaluated using the chi-square test of independence. Crude logistic regression models were used to estimate unadjusted odds ratios (ORs) and 95% confidence intervals. A *p*-value < 0.05 was considered statistically significant. Additionally, an exploratory analysis was conducted to evaluate clinical factors associated with the type of benzodiazepine prescribed. Variables available in the dataset included polypharmacy, sleep disorders, and cognitive status (MMSE categories). Due to the presence of low-frequency categories in the outcome variable, benzodiazepine type was dichotomized into the most frequently prescribed category versus other types to improve model stability. Associations were initially explored using chi-square tests, followed by a logistic regression model to estimate crude odds ratios (ORs) and 95% confidence intervals.

### 2.6. Ethical Considerations

The study was conducted in accordance with the principles of the Declaration of Helsinki and the Mexican General Health Law. The research protocol was reviewed and approved by the Local Research and Ethics Committees of the HGZ MF No. 2 (registration number R-2025-1003-002).

All participants received verbal and written information about the study and provided written informed consent prior to enrollment. Participant confidentiality was strictly maintained, and all data were analyzed anonymously for research purposes only.

## 3. Results

### 3.1. Study Population Characteristics

Detailed sociodemographic characteristics are presented in Table 1. A total of 228 older adults aged 60 years and older with documented benzodiazepine use were included in the analysis. Women accounted for 65.4% (*n* = 149) of the participants, and men for 34.6% (*n* = 79). The most frequent age group was 60–69 years (39.0%), followed by 70–79 years (35.1%), 80–89 years (22.4%), and ≥90 years (3.1%).

Regarding educational attainment, primary school education was the most common level (46.0%), followed by secondary education (26.3%). A total of 12.0% of participants were illiterate. Detailed sociodemographic characteristics are shown in Table 1.

### 3.2. Educational Level and Cognitive Impairment

Educational level was significantly associated with cognitive status (χ^2^ = 17.41; *p* = 0.026). Participants with lower educational attainment, particularly those with primary education or no formal education, accounted for a larger proportion of cases of mild/moderate and major cognitive impairment (Table 2).

### 3.3. Cognitive Status

Based on MMSE assessment, normal cognitive performance was observed in 38.2% (n = 87) of participants. Mild to moderate cognitive impairment was observed in 36.8% (n = 84), while major cognitive impairment was observed in 25.0% (n = 57).

### 3.4. Benzodiazepine Use and Duration

Clonazepam was the most frequently prescribed benzodiazepine. Long-acting benzodiazepines predominated over short-acting agents in the study population. In terms of duration of use, 56.6% of participants had used benzodiazepines for 3–6 years, 22.4% for less than 3 years, and 21.1% for more than 6 years.

### 3.5. Association Between Duration of Benzodiazepine Use and Cognitive Impairment

A statistically significant association was observed between duration of benzodiazepine use and cognitive status. (χ^2^ = 25.81; *p* < 0.001). Participants with longer durations of benzodiazepine use showed a higher proportion of cognitive impairment.

Specifically, the highest proportion of major cognitive impairment was observed among individuals with benzodiazepine use exceeding six years. The distribution of cognitive status according to duration of use is presented in Table 3.

### 3.6. Logistic Regression Analysis

When cognitive impairment was analyzed as a binary outcome, longer duration of benzodiazepine use was associated with higher odds of impairment. Compared with <3 years, participants with 3–6 years (OR 2.43; 95% CI 1.26–4.71; *p* = 0.008) and >6 years (OR 2.43; 95% CI 1.08–5.50; *p* = 0.032) showed increased odds. These estimates are unadjusted and should be interpreted cautiously. These estimates correspond to crude (unadjusted) models and should be interpreted as exploratory, as no adjustment for major confounding variables was performed.

### 3.7. Additional Exploratory Analysis of Clinical Factors

The associations between clinical variables and benzodiazepine type are presented in Table 4. In an exploratory analysis, additional clinical variables derived from the dataset were evaluated to identify factors associated with the type of benzodiazepine prescribed. Due to the distribution of the outcome variable and the presence of low-frequency categories, the benzodiazepine type was dichotomized into the most frequently prescribed category versus other types, allowing a more robust analysis.

Chi-square tests demonstrated significant associations between benzodiazepine prolonged use > 24 h and the following variables:

Sleep disorders (χ^2^ = 11.56; *p* = 0.009);

Polypharmacy (χ^2^ = 10.28; *p* = 0.016);

Mini-Mental State Examination (MMSE) score category (χ^2^ = 13.87; *p* = 0.031).

No significant associations were observed with sex, educational level, alcohol use, or duration of benzodiazepine use in this analysis.

Results from the logistic regression model are summarized in Table 5. A logistic regression model was constructed using these variables. In the adjusted model:

Polypharmacy was associated with increased odds of receiving the most frequently prescribed benzodiazepine (OR 2.37; 95% CI 1.27–4.41; *p* = 0.007).

Sleep disorders were also associated with higher odds (OR ≈ 2.10; *p* < 0.01).

Cognitive status based on MMSE showed a significant association (*p* = 0.031), indicating that cognitive impairment influenced prescribing patterns.

These findings suggest that benzodiazepine prescribing in this population is influenced primarily by clinical factors, particularly cognitive status, sleep disorders, and medication burden.

## 4. Discussion

This study describes patterns of cognitive impairment among benzodiazepine users rather than evaluating causal associations. In this cross-sectional study conducted in a primary care setting, longer duration of benzodiazepine exposure was associated with worse cognitive status among users. These findings are consistent with a substantial body of international evidence reporting associations between prolonged benzodiazepine exposure and adverse cognitive outcomes, reinforcing longstanding concerns regarding their chronic prescription in aging populations [8,9,10,11,16,17,18].

The prevalence of cognitive impairment observed among benzodiazepine users in this study (61.8%) was notably higher than that reported in general community-dwelling older populations. Global epidemiological estimates indicate that the prevalence of cognitive impairment among adults aged 60 years and older ranges from approximately 2% to 8.5%, depending on region, educational attainment, and assessment methods [1,2,16]. This discrepancy suggests that older adults receiving benzodiazepines in primary care may represent a particularly vulnerable subgroup characterized by a higher burden of neuropsychiatric symptoms, multimorbidity, and pharmacological exposure.

A central finding of the present study is the observed association between duration of benzodiazepine use and severity of cognitive impairment. Although crude analyses suggested higher odds of impairment with longer duration of use, these estimates must be interpreted cautiously. Given the absence of multivariable adjustment, the magnitude of the reported odds ratios should not be interpreted as independent effects, and residual confounding cannot be excluded. This duration-response pattern is in line with previous longitudinal and observational studies reporting stronger associations with long-term or cumulative benzodiazepine use compared with short-term exposure [9,11,16,17,18]. These findings support the notion that chronic exposure may be an important marker of cognitive vulnerability in older adults.

An additional relevant finding of this study is that benzodiazepine prescribing patterns were significantly associated with clinical factors, including polypharmacy, sleep disorders, and cognitive status. These results suggest that prescribing decisions are not random but rather guided by patient-specific clinical characteristics. The association with polypharmacy may reflect cautious selection of benzodiazepines to minimize drug interactions, while the influence of sleep disorders is consistent with the primary indication for these medications. Furthermore, the relationship with cognitive status highlights the complexity of prescribing in older adults, where clinicians may tailor pharmacological choices based on cognitive vulnerability. This pattern supports the notion of a clinically driven and individualized prescribing approach in primary care settings.

Confounding by indication represents an important consideration in interpreting these findings. Benzodiazepines are commonly prescribed for conditions such as anxiety, insomnia, and depression, which themselves have been independently associated with cognitive impairment and increased risk of dementia [5,6,12,16,17,18]. Consequently, it is possible that part of the observed association reflects underlying neuropsychiatric conditions rather than benzodiazepine exposure alone. The cross-sectional design of the study precludes establishing temporal relationships, and reverse causation cannot be excluded, as early cognitive symptoms may prompt benzodiazepine prescription. Therefore, benzodiazepine exposure should be interpreted as a marker of underlying clinical conditions rather than an independent causal factor.

Educational level was also significantly associated with cognitive status in this cohort, with lower educational attainment linked to more severe cognitive impairment. This finding is consistent with the cognitive reserve hypothesis and with previous population-based studies demonstrating higher prevalence and earlier onset of cognitive impairment among individuals with limited formal education, particularly in middle- and low-income countries [2,19]. Educational level may additionally influence performance on cognitive screening instruments such as the MMSE, potentially amplifying observed associations in primary care settings [3,20,21].

From a clinical and safety perspective, these findings align with recommendations from the American Geriatrics Society Beers Criteria^®^, which classify benzodiazepines as potentially inappropriate medications for older adults due to their association with cognitive impairment, delirium, falls, and fractures [7]. Despite these recommendations, benzodiazepines remain widely prescribed in primary care, often for prolonged periods. The predominance of clonazepam observed in this study mirrors prescribing patterns reported in other settings and is noteworthy given its pharmacokinetic profile and central nervous system effects. Clonazepam is a long-acting benzodiazepine with a prolonged half-life, which may increase the risk of accumulation and central nervous system effects in older adults [5,6,22].

Emerging neuroimaging evidence provides additional biological plausibility for the observed associations. Population-based studies have reported associations between benzodiazepine use and reduced volumes in brain structures involved in cognition, including the hippocampus and thalamus, as well as accelerated brain atrophy over time [12]. Although these findings do not establish causality, they support concerns regarding long-term benzodiazepine exposure and cognitive vulnerability.

In light of these findings, deprescribing strategies in primary care warrant particular attention. Evidence-based interventions have demonstrated that structured deprescribing approaches can reduce long-term benzodiazepine use among older adults without worsening underlying symptoms, particularly when combined with non-pharmacological alternatives [23,24,25,26]. Integrating regular medication review and deprescribing protocols into routine primary care practice may represent a feasible strategy to mitigate potentially preventable cognitive harm.

This study has several limitations. Its cross-sectional design precludes causal inference, and the absence of multivariable adjustment limits control for potential confounders such as psychiatric comorbidities, sleep disorders, alcohol use, and polypharmacy. Additionally, the inability to distinguish between current and past benzodiazepine use may have introduced exposure misclassification. Cognitive status was assessed using the MMSE, which is influenced by educational level and may lack sensitivity for early cognitive impairment [3,20,21]. Additionally, data were derived from a single primary care center, which may limit generalizability.

Despite these limitations, this study provides relevant real-world evidence from a primary care population in a middle-income country, addressing an important gap in the literature. The findings highlight benzodiazepine use—particularly long-term use—as an important marker of cognitive vulnerability in older adults and support initiatives aimed at safer prescribing, regular medication review, and deprescribing strategies in primary care. The crude regression analysis does not account for important confounders, and the magnitude of associations may therefore be overestimated. Detailed information on dosage, cumulative exposure, treatment indication, and concomitant medications was not available.

## 5. Conclusions

Among benzodiazepine users, longer duration of exposure was associated with worse cognitive status. These findings should be interpreted as descriptive and exploratory and do not establish causal relationships.

Additionally, benzodiazepine prescribing patterns were associated with clinical factors such as polypharmacy, sleep disorders, and cognitive status, suggesting that prescribing decisions in primary care are largely guided by individualized clinical characteristics.

Overall, these findings highlight benzodiazepine use—particularly prolonged exposure—as an important marker of cognitive vulnerability in older adults attending primary care units. They underscore the need for careful long-term prescribing practices, regular medication review, and consideration of deprescribing strategies, in accordance with current geriatric prescribing recommendations.

This study provides relevant real-world evidence from a primary care population in a middle-income country, contributing to the understanding of benzodiazepine use and cognitive health in routine clinical practice. Further longitudinal studies with multivariable adjustment are warranted to clarify temporal relationships and to better delineate the independent contribution of benzodiazepine exposure to cognitive outcomes in older adults.

## Figures and Tables

**Table 1:** Sociodemographic characteristics of the study population (n = 228).

Variable	Category	n	%
Sex	Female	149	65.4
	Male	79	34.6
Age group (years)	60–69	90	39.0
	70–79	80	35.1
	80–89	51	22.4
	≥90	7	3.1
Educational level	Illiterate	27	12.0
	Primary school	104	46.0
	Secondary school	60	26.3
	High school	23	10.1
	University degree	14	6.1

Data are presented as absolute frequency (n) and percentage (%).

**Table 2:** Association between educational level and cognitive status.

Educational Level	Normal (%)	Mild/Moderate Impairment (%)	Major Impairment (%)	Total (%)
Illiterate	14 (16.1)	10 (11.9)	3 (5.3)	27 (12.0)
Primary school	34 (39.1)	40 (47.6)	30 (52.6)	104 (46.0)
Secondary school	17 (19.5)	23 (27.4)	20 (35.1)	60 (26.3)
High school	12 (13.8)	8 (9.5)	3 (5.3)	23 (10.1)
University degree	10 (11.5)	3 (3.6)	1 (1.7)	14 (6.1)
Total	87 (100)	84 (100)	57 (100)	228 (100)

Data are presented as absolute frequency (n) and percentage (%). Chi-square test: χ^2^ = 17.41; *p* = 0.026.

**Table 3:** Association between duration of benzodiazepine use and cognitive status (n = 228).

Cognitive Status	<3 Years (%)	3–6 Years (%)	>6 Years (%)	Total (%)
Normal	28 (54.9)	43 (33.3)	16 (33.3)	87 (38.2)
Mild/Moderate impairment	16 (31.4)	59 (45.7)	9 (18.8)	84 (36.8)
Major impairment	7 (13.7)	27 (21)	23 (47.9)	57 (25.0)
Total	51 (100)	129 (100)	48 (100)	228 (100)

Data are presented as absolute frequency (n) and percentage (%). Chi-square test: χ^2^ = 25.81; *p* < 0.001.

**Table 4:** Association between clinical factors and benzodiazepine type.

Variable	Category	Prolonged Use > 24 h	Short- < 6 h, Intermediate-Use 6 to 24 h	*p*-Value
Polifarmacia	Yes	81 (65.3)	43 (34.74)	0.016
	No	85 (81.7)	19 (18.3)	
Sleep disorders	Yes	130 (68.4)	60 (31.6)	0.009
	No	36 (94.7)	2 (5.3)	
MMSE category	Normal	67 (77.0)	20 (23.0)	0.031
	Mild/moderate	61 (67.8)	29 (32.2)	
	Major	38 (74.5)	13 (25.5)	

Data are presented as absolute frequency (n) and percentage (%).

**Table 5:** Logistic regression analysis.

Variable	OR	95% CI	*p*-Value
Polifarmacia	2.37	1.27–4.41	0.007
Sleep disorders	2.10	1.10–3.90	0.009
MMSE category	1.85	1.05–3.20	0.031

Odds ratios (OR) and 95% confidence intervals (CI).

## Data Availability

The data supporting the findings of this study are not publicly available due to privacy and ethical restrictions, as they contain sensitive information related to human participants. The datasets generated during the current study are available from the corresponding author upon reasonable request.

## References

[1] Prince M., Wimo A., Guerchet M., Ali G.C., Wu Y.-T., Prina M. (2015). World Alzheimer Report 2015: The Global Impact of Dementia.

[2] Li Z., Gong X., Wang S., Liu M., Liu S., Wang Y., Wu D., Yang M., Li R., Li H. (2022). Cognitive impairment assessed by Mini-Mental State Examination predicts all-cause and CVD mortality in Chinese older adults: A 10-year follow-up study. Front. Public Health.

[3] Tortora C., Teixeira L., Sousa S., Paúl C. (2025). MMSE in primary care practice: Why good tests can mislead in the wrong context. Eur. J. Ageing.

[4] Salis F., Costaggiu D., Mandas A. (2023). Mini-Mental State Examination: Optimal cut-off levels for mild and severe cognitive impairment. Geriatrics.

[5] Markota M., Rummans T.A., Bostwick J.M., Lapid M.I. (2016). Benzodiazepine use in older adults: Dangers, management, and alternative therapies. Mayo Clin. Proc..

[6] Danza Á., Cristiani F., Tamosiunas G. (2009). Risks associated with benzodiazepine use. Arch. Med. Interna.

[7] 2023 American Geriatrics Society Beers Criteria^®^ Update Expert Panel (2023). American Geriatrics Society 2023 updated AGS Beers Criteria^®^ for potentially inappropriate medication use in older adults. J. Am. Geriatr. Soc..

[8] Islam M.M., Iqbal U., Walther B., Atique S., Dubey N.K., Nguyen P.-A., Poly T.N., Masud J.H.B., Li Y.-C., Shabbir S.-A. (2016). Benzodiazepine use and risk of dementia in the elderly population: A systematic review and meta-analysis. Neuroepidemiology.

[9] Liu L., Jia L., Jian P., Zhou Y., Zhou J., Wu F., Tang Y. (2020). The effects of benzodiazepine use and abuse on cognition in the elderly: A systematic review and meta-analysis. Front. Psychiatry.

[10] Wu C.-C., Liao M.-H., Su C.-H., Poly T.N., Lin M.-C. (2023). Benzodiazepine use and the risk of dementia in the elderly population: An umbrella review of meta-analyses. J. Pers. Med..

[11] Paterniti S., Dufouil C., Alpérovitch A. (2002). Long-term benzodiazepine use and cognitive decline in the elderly: The Epidemiology of Vascular Aging Study. J. Clin. Psychopharmacol..

[12] vom Hofe I., Stricker B.H., Vernooij M.W., Ikram M.A., Wolters F.J. (2024). Benzodiazepine use in relation to long-term dementia risk and imaging markers of neurodegeneration. BMC Med..

[13] Montes-Castrejón A., Moncayo-Samperio L.G., Flores-Ramos M. (2024). Benzodiazepine Consumption, Functionality, Cognition, and Somnolence in Older Adults at a Tertiary Care Psychiatric Hospital in Mexico City. Cureus.

[14] Baek M.J., Kim K., Park Y.H., Kim S. (2016). The Validity and Reliability of the Mini-Mental State Examination-2 for Detecting Mild Cognitive Impairment and Alzheimer’s Disease in a Korean Population. PLoS ONE.

[15] Maduro P.A., Carvalho L.P.C., Maduro L.A.R., Rodrigues A.B.C., Rocha A.S.L., Matos L.R.R.S., Nascimento M.M., Gambassi B.B., Schwingel P.A. (2025). Accuracy of the Mini-Mental State Examination and Montreal Cognitive Assessment in Detecting Cognitive Impairment in Older Adults: A Comparative Study Adjusted for Educational Level. NeuroSci.

[16] Billioti de Gage S., Moride Y., Ducruet T., Kurth T., Verdoux H., Tournier M., Pariente A., Bégaud B. (2014). Benzodiazepine use and risk of Alzheimer’s disease: Case-control study. BMJ.

[17] Gray S.L., Dublin S., Yu O., Walker R., Anderson M., Hubbard R.A., Crane P.K., Larson E.B. (2016). Benzodiazepine use and risk of incident dementia or cognitive decline: Prospective population based study. BMJ.

[18] Pariente A., de Gage S.B., Moore N., Bégaud B. (2016). The benzodiazepine–dementia disorders link: Current state of knowledge. CNS Drugs.

[19] Arevalo-Rodriguez I., Smailagic N., Roqué-Figuls M., Ciapponi A., Sanchez-Perez E., Giannakou A., Pedraza O.L., Cosp X.B., Cullum S. (2021). Mini-Mental State Examination for the early detection of dementia in people with mild cognitive impairment. Cochrane Database Syst. Rev..

[20] Reeve E., Ong M., Wu A., Jansen J., Petrovic M., Gnjidic D. (2017). A systematic review of interventions to deprescribe benzodiazepines and other hypnotics among older people. Eur. J. Clin. Pharmacol..

[21] Pottie K., Thompson W., Davies S., Grenier J., Sadowski C.A., Welch V., Holbrook A., Boyd C., Swenson R., Farrell A.M.B. (2018). Deprescribing benzodiazepine receptor agonists: Evidence-based clinical practice guideline. Can. Fam. Physician.

[22] Seppala L.J., van der Velde N., Masud T., Blain H., Petrovic M., van der Cammen T.J., Szczerbińska K., Hartikainen S., Kenny R.A., Ryg J. (2019). EuGMS Task and Finish group on Fall-Risk-Increasing Drugs (FRIDs): Position on Knowledge Dissemination, Management, and Future Research. Drugs Aging.

[23] Bloch F., Thibaud M., Dugué B., Brèque C., Rigaud A.-S., Kemoun G. (2011). Psychotropic drugs and falls in the elderly people: Updated literature review and meta-analysis. J. Aging Health.

[24] Mitchell A.J. (2009). A meta-analysis of the accuracy of the Mini-Mental State Examination in the detection of dementia. J. Psychiatr. Res..

[25] Livingston G., Huntley J., Liu K.Y., Costafreda S.G., Selbaek G., Alladi S., Ames D., Banerjee S., Burns A., Brayne C. (2024). Dementia prevention, intervention, and care: 2024 report of the Lancet standing Commission. Lancet.

[26] World Health Organization (2019). Risk Reduction of Cognitive Decline and Dementia: WHO Guidelines.

